# A Giant Vulvar Mass: A Case Study of Cellular Angiofibroma

**DOI:** 10.1155/2016/2094818

**Published:** 2016-05-16

**Authors:** Ümit Aydın, Hasan Terzi, Ünal Turkay, Ahmet Tuğrul Eruyar, Ahmet Kale

**Affiliations:** ^1^Obstetrics and Gynecology Clinic, Kocaeli Derince Education and Research Hospital, 41900 Kocaeli, Turkey; ^2^Department of Pathology, Kocaeli Derince Education and Research Hospital, 41900 Kocaeli, Turkey

## Abstract

Cellular angiofibroma is a mesenchymal tumor that affects both genders. Nucci et al. first described it in 1997. Cellular angiofibroma is generally a small and asymptomatic mass that primarily arises in the vulvar-vaginal region, although rare cases have been reported in the pelvic and extrapelvic regions. It affects women most often during the fifth decade of life. The treatment requires simple local excision due to low local recurrence and no chance of metastasization. The current study presents a case of angiofibroma in the vulvar region that measured approximately 20 cm.

## 1. Introduction

Cellular angiofibroma is a soft tissue tumor seen in the vulvar-vaginal region in women and in the scrotum inguinal region in men [1, 2]; it is rarely reported to have extragenital localization [3–5]. In 1997, Nucci et al. [1] described six cases of cellular angiofibroma in middle-aged women. Later, Laskin et al. [2] reported cases with similar histological characteristics in the scrotal-inguinal region of 11 male patients. The tumors are well-circumscribed, superficial, and soft tissue-localized and consist of soft spindle cells and small- and medium-sized veins with mural hyalinization [6].

In the cellular angiofibroma cases described in the literature, the size of the tumors is reported to be between 0.6 and 12.3 cm, with an average of 3.6 cm. To the best of our knowledge, the present case involves the largest vulvar cellular angiofibroma reported in the literature.

## 2. Case Representation

**Figure 1:**
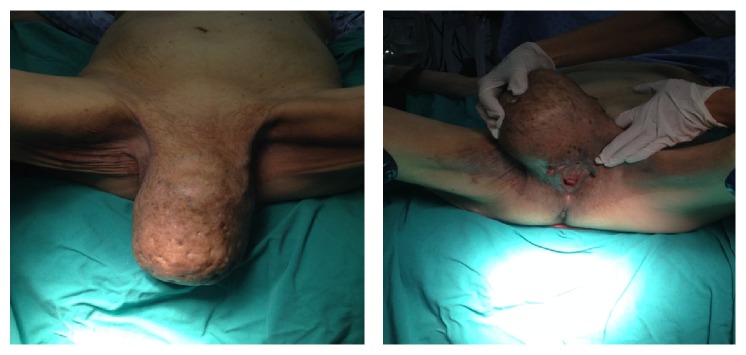
Angiofibroma arising from the right labia major.

A 59-year-old female patient presented with complaints of a mass of approximately 20 × 15 × 10 cm that was well-circumscribed, obvious, and solid, completely covering the right labium majus and extending to the vulva and 1/3 of the femur (Figure 1). The patient history revealed that the mass was first identified five years earlier but had grown rapidly within the previous year. The mass was painless but caused discomfort when sitting or walking due to its size. There was no family history of gynecological cancer. She did not use any hormone replacement therapy. The patient's gynecological examination was normal, and she had been in the postmenopausal period for ten years. No palpable inguinal lymph nodes were determined. Using USG, the inguinal hernia was excluded and the Magnetic Resonance Imaging (MRI) evaluation reported a mesenchymal soft tissue tumor. The hematological and biochemical parameters of the case were normal.

**Figure 2:**
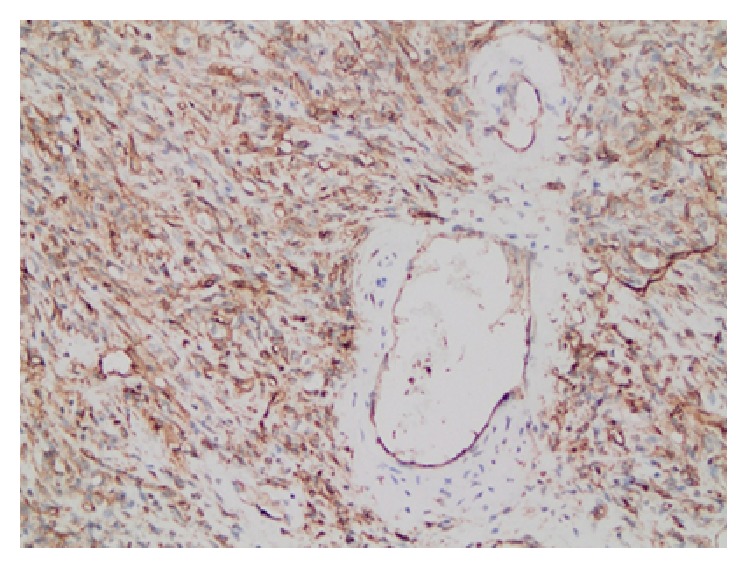
Diffuse and strong expression for CD34 were observed in cytoplasm of spindle-shaped tumor cells. CD34 ×100.

The patient underwent surgery with a diagnosis of a mass in the vulva; the mass was easily excised through a skin incision, because the pseudocapsule was easily enucleated. In the histopathological examination, the tumor tissue was found to be relatively elastic, encapsulated, and grey-white with a lobulated appearance. Immunohistochemical examination showed positivity in the CD34 and tumor and in the SMA and vascular structures (Figure 2). Focal positivity with desmin, estrogen, and progesterone and positivity in vascular structures were observed with CD31. The proliferation index of the tumor was approximately 3–5% in three different areas, which was assessed with KI67. Patient was followed up for 6 months and there was no recurrence.

## 3. Discussion

Cellular angiofibroma is a small, well-circumscribed, mostly asymptomatic, typically slow growing, and benign stromal tumor that has been diagnosed equally in male and female patients in recent years and most often found in the distal genital region. In women, it is frequently observed in the fifth decade (average age: 46.1 years) and in men in the seventh decade.

Mandato et al. [7] investigated the literature on 79 female patients between 20 and 77 years of age. Most of the cases occurred in the vulvar-vaginal region, but in five patients the localization was pelvic and in four patients the localization was perineal. Six cases had extrapelvic, left hip, knee, thorax axilla, breast, and hypochondrium localization, respectively [3, 5]; one postmenopausal patient had paravesical area localization [8]. Tumor sizes were between 0.6 and 12.3 cm, and diagnosis was made before surgery in only 25 cases. The present case is the largest cellular angiofibroma defined in the literature, measuring 20 cm. In the clinical assessment, inguinal hernia must be considered as a differential diagnosis. Cellular angiofibroma was confused with Bartholin's cyst in 48% of the cases, with a nonspecific solid mass in 28% of the cases, with vulvar cyst in 12% of the cases, with leiomyoma in 8% of the cases, and with lipoma in 4% of cases. In the present case, hernia was excluded after the examination, dimensions, and USG assessment.

All 79 cases summarized by Mandato et al. [7] were treated with simple resection, and the surgical boundaries were not defined in 47 cases; the surgical boundary was positive in 18 cases and negative in 29 cases. In the follow-up, five cases with positive surgical boundaries required reexcision [7]. In the present case, simple surgical resection was performed and the surgical boundaries were reported to be histopathologically negative.

Of the 79 cases summarized by Mandato et al. [7], 48 were followed up after 3–240 months; local recurrence and metastasis were not observed, not even in the atypical and sarcomatous transformation group. In another study of 12 patients, no recurrence or metastasis was reported at the 14-month follow-up [6].

One case of cellular angiofibroma had only a sarcomatous component, which was excised 27 months after the diagnosis of cellular angiofibroma due to metastatic carcinoma of unknown primary and 59 months after the diagnosis of cellular angiofibroma due to breast cellular angiofibroma [6].

In the differential diagnosis, spindle cell lipoma, angiomyofibroblastoma, aggressive angiomyxoma, and smooth muscle tumor must be considered [9]. In the differential diagnosis of soft tissue tumors, immunohistochemical characteristics are useful. Immunohistochemistry is also useful in indicating atypical and sarcomatous transformation.

The expression of estrogen and progesterone receptors is very interesting in cellular angiofibroma. The fact that cellular angiofibroma is present in the menopausal and postmenopausal periods suggests that pathogenic development may be hormonally influenced. In the present case, the estrogen and progesterone receptors were positive. However, the pathogenesis of cellular angiofibroma remains unclear. Although sexual hormones and Reactive Oxygen Species (ROS) are possible factors, HPV E7 oncoprotein has also been suggested, but no adequate studies have been conducted [10].

Cellular angiofibroma is considered benign. No metastasis has been reported in the literature, and recurrence was reported in only one case [11]. In the reported cases, simple local excision was adequate, and in cases with atypical and sarcomatous transformation, radical excision with negative surgical boundaries is recommended [6, 11]. In addition, in five of the 18 patients who had positive surgical boundaries, local recurrence was realized and excision was adequate. In the remaining 13 cases with positive boundaries, recurrence or metastasis was not observed [7]. Thus, broad surgical excision is not recommended in cellular angiofibroma cases.

The present case involves the largest vulvar cellular angiofibroma reported in the literature. Cellular angiofibroma should be considered in the differential diagnosis of painless soft masses that may reach large dimensions in the vulva. Although it is a benign tumor and shows different phenotypic characteristics, simple local excision is adequate, because it demonstrates no recurrence or metastasis.
